# Proton Therapy for HPV-Associated Oropharyngeal Cancers of the Head and Neck: a De-Intensification Strategy

**DOI:** 10.1007/s11864-021-00847-y

**Published:** 2021-06-04

**Authors:** Nicolette Taku, Li Wang, Adam S. Garden, David I. Rosenthal, G. Brandon Gunn, William H. Morrison, C. David Fuller, Jack Phan, Jay P. Reddy, Amy C. Moreno, Michael T. Spiotto, Gregory Chronowski, Shalin J. Shah, Lauren L. Mayo, Neil D. Gross, Renata Ferrarotto, X. Ronald Zhu, Xiaodong Zhang, Steven J. Frank

**Affiliations:** 1 Department of Radiation Oncology, The University of Texas MD Anderson Cancer Center, 1515 Holcombe Boulevard, Houston, TX 77030 USA; 2 Department of Experimental Radiation Oncology, The University of Texas MD Anderson Cancer Center, Houston, TX USA; 3 Department of Head and Neck Surgery, The University of Texas MD Anderson Cancer Center, Houston, TX USA; 4 Department of Head and Neck Medical Oncology, The University of Texas MD Anderson Cancer Center, Houston, TX USA; 5 Department of Radiation Physics, The University of Texas MD Anderson Cancer Center, Houston, TX USA

**Keywords:** Head and neck, Proton therapy, De-intensification, Oropharyngeal cancer, Clinical trials, De-escalation

## Abstract

The rise in the incidence of human papillomavirus (HPV)-associated oropharyngeal squamous cell carcinoma (OPC), the relatively young age at which it is diagnosed, and its favorable prognosis necessitate the use of treatment techniques that reduce the likelihood of side effects during and after curative treatment. Intensity-modulated proton therapy (IMPT) is a form of radiotherapy that de-intensifies treatment through dose de-escalation to normal tissues without compromising dose to the primary tumor and involved, regional lymph nodes. Preclinical studies have demonstrated that HPV-positive squamous cell carcinoma is more sensitive to proton radiation than is HPV-negative squamous cell carcinoma. Retrospective studies comparing intensity-modulated photon (X-ray) radiotherapy to IMPT for OPC suggest comparable rates of disease control and lower rates of pain, xerostomia, dysphagia, dysgeusia, gastrostomy tube dependence, and osteoradionecrosis with IMPT—all of which meaningfully affect the quality of life of patients treated for HPV-associated OPC. Two phase III trials currently underway—the “Randomized Trial of IMPT versus IMRT for the Treatment of Oropharyngeal Cancer of the Head and Neck” and the “TOxicity Reduction using Proton bEam therapy for Oropharyngeal cancer (TORPEdO)” trial—are expected to provide prospective, level I evidence regarding the effectiveness of IMPT for such patients.

## Introduction

With a projected incidence of 65,000 cases in 2020, cancers of the head and neck (HNC) constitute approximately 3% of malignancies in the USA [1]. External beam radiotherapy (EBRT) is important in the management of HNC, with 75% of HNC patients undergoing EBRT as either primary or postoperative therapy [2]. Although the overall incidence of HNC has declined in recent decades, the incidence of oropharyngeal cancer (OPC) associated with human papillomavirus (HPV) infection has increased, with 70-90% of newly diagnosed OPC cases showing molecular findings consistent with HPV positivity [3]. Patients with HPV-associated OPC are more likely to be diagnosed in the 4th or 5th decade of life and have a more favorable prognosis than patients with smoking-associated (HPV-negative) cancers—underscoring the need for precise, conformal radiotherapy that minimizes the likelihood of long-term, treatment-related side effects.

## HPV-associated oropharyngeal cancer

Approximately 15,800 cases of HPV-associated OPC are diagnosed annually in the USA [4]. Transmitted via orogenital contact, HPV infects the basal epithelial layer of the oropharyngeal mucosa and integrates itself into the host genome. The HPV oncoproteins E6 and E7 bind and degrade the host p53 and Rb tumor suppressor proteins, respectively, inhibit cell death pathways, and promote cellular proliferation [5]. Infection with high-risk HPV subtypes, of which HPV16 is the most common, can result in cancers of the tonsils and base of the tongue. HPV-associated cancers of the other oropharyngeal subsites (uvula, soft palate, and posterior pharyngeal wall) are less common.

Some early-stage HPV-associated OPC, particularly in “never-smokers” or smokers with a less than 10-pack-year history, can be managed with surgery or EBRT [6, 7]. However, because disease often presents with extensive lymphadenopathy or radiographic evidence of extranodal extension, definitive chemoradiotherapy or combined treatment with surgery followed by adjuvant radiotherapy (with or without chemotherapy) is the mainstay of treatment [8]. Consensus guidelines recommend radiation doses of 66–70 Gy for definitive treatment and 60–66 Gy for postoperative radiation [9].

For patients receiving chemoradiotherapy, cisplatin is the agent of choice over other systemic agents, including cetuximab. Trial 1016 from the Radiotherapy Oncology Group (RTOG) was a treatment de-intensification study with a non-inferiority design that compared EBRT given with either the alkylating chemotherapy agent cisplatin or the epidermal growth factor receptor (EGFR)-neutralizing antibody cetuximab, with the hypothesis that concurrent cetuximab would yield high rates of patient survival and lower rates of treatment-related toxicity [10]. At a median interval of 4.5 years of follow-up, clinical outcomes for cetuximab concurrent with radiotherapy failed to meet the predetermined non-inferiority criterion, in that the 5-year overall survival rate for patients given concurrent cetuximab (77.9%; 95% confidence interval (CI) 73.4–82.5) was lower than that achieved with concurrent cisplatin (84.6%; 95% CI 80.6–88.6). Progression-free survival and locoregional control rates were also worse with cetuximab. However, rates of moderate to severe acute and late side effects were comparable between the two treatment groups [10]. Similar outcomes were observed in the De-ESCALaTE HPV study, a phase III trial comparing chemoradiotherapy with cisplatin versus cetuximab for low-risk (i.e., never-smokers or smokers with a less than 10-pack-year history) HPV-associated OPC, and ARTSCAN III, a randomized phase III trial comparing chemoradiotherapy with cisplatin versus cetuximab for locoregionally advanced squamous cell carcinoma of the head and neck [11, 12].

## Radiotherapy treatment side effects

As is true for chemotherapy, considerable effort has been dedicated to minimizing the side effects of EBRT experienced during and after treatment for OPC. Radiation-induced side effects are directly related to the doses received by normal tissues, including the salivary glands, pharyngeal constrictor muscles, mandible, spinal cord, cochlea/vestibular apparatus, and brainstem [13]. The development of multi-leaf collimators greatly advanced the conformality of radiation doses. Composed of sliding metal leaflets, multi-leaf collimators modulate the intensity of a radiation beam, thereby allowing clinicians to use either static, discrete radiation fields (intensity-modulated photon (X-ray) radiotherapy (IMRT)) or dynamic, contiguous arcs (volumetric arc radiotherapy (VMAT)) to “sculpt” the dose around the areas of interest [14, 15].

The ability of IMRT and VMAT to sculpt the radiation beam and thereby reduce the volume of high-dose radiation delivered to normal, non-targeted tissues has reduced the occurrence of several radiation-related side effects, including xerostomia and osteoradionecrosis. A meta-analysis of HNC studies comparing 2-dimensional and 3-dimensional (2D and 3D) radiation treatment techniques to IMRT found a 25% improvement in grade 2-4 xerostomia (hazard ratio = 0.76, 95% CI 0.66–0.87, *P*<0.0001) with IMRT [16]. When used with prophylactic dental care, IMRT and VMAT have also led to substantial decreases in the incidence of osteoradionecrosis, the risk of which in modern studies has been estimated at less than 5% [17, 18]. Although dysphagia continues to be a common side effect of radiotherapy, the lower mean doses to the uninvolved pharyngeal constrictor muscles and larynx that are achieved with IMRT and VMAT techniques have improved both the incidence and severity of symptoms [19].

Despite their ability to deliver conformal, high-dose radiation to targeted regions of the head and neck, IMRT and VMAT conversely distribute lower-dose radiation to a greater volume of non-targeted structures [20]. This collateral dose can have adverse clinical consequences. Rosenthal et al. demonstrated that, in the treatment of OPC, radiation dose to normal tissues located along the IMRT beam path resulted in worsening of several side effects relative to 3D conformal radiotherapy (3D-CRT), including high-grade oral mucositis [21]. Oral mucositis is associated with significant pain, dysphagia, weight loss, feeding tube placement, and unplanned hospitalizations. Severe mucositis, particularly when it results in treatment interruptions, can adversely influence disease control [22].

## Radiotherapy dose de-escalation

Given the side effects associated with EBRT, radiation dose de-escalation has been investigated as a way of maintaining rates of disease control for HPV-associated OPC while reducing treatment-related morbidity [23]. Chera et al. studied patients with T0-T3, N0-N2c, M0 disease (according to the 7th edition of the American Joint Committee on Cancer (AJCC) staging system) who were treated with reduced-dose definitive chemoradiotherapy consisting of 60 Gy with weekly cisplatin (30 mg/m^2^). Surgical evaluation performed at 7-14 weeks after treatment completion demonstrated pathologic complete response rates of 98% for the primary site and 84% for involved lymph nodes [24]. Because pathologic response is a surrogate for clinical outcomes, this study served as proof of principle that reduced-dose radiation as definitive therapy may achieve favorable rates of locoregional control.

Several studies have also investigated outcomes for patients with HPV-associated OPC treated with de-escalated radiation doses after surgery [25–27]. The Eastern Cooperative Oncology Group (ECOG) and the American College of Radiology Imaging Network (ACRIN) E3311 trial enrolled patients who were to receive transoral resection of the primary disease and neck dissection of lymph nodes. Those patients found postoperatively to be at intermediate risk of disease recurrence because of close margins at the primary site, evidence of perineural invasion/lymphovascular invasion, metastatic involvement of 2 to 4 lymph nodes, or metastatic involvement of least 1 lymph node and no more than 1 mm of extranodal extension were randomly assigned to receive either 50 Gy or 60 Gy of postoperative radiation [28•]. At a median follow-up interval of 31.8 months, the progression-free survival rates were 95.0% (90% CI 91.4–98.6) for those given 50 Gy and 95.9% (90% CI 92.6–99.3) for those given 60 Gy. Additional studies are needed, but the results of this randomized phase II trial support the use of lower radiation doses for postoperative therapy in at least some instances of HPV-associated OPC.

Although induction chemotherapy is not considered part of standard of care for HPV-associated OPC, several trials have been conducted to evaluate induction chemotherapy followed by reduced-dose radiotherapy for those showing clinical response to the induction therapy [29–32]. The ECOG-ACRIN E1308 trial reported outcomes for patients with resectable, stage III or IV, HPV-associated OPC (per AJCC 7th edition) who received 3 cycles of induction chemotherapy followed by chemoradiotherapy to 54 Gy for those with clinical complete response at the primary site of disease. Among complete responders, the 2-year progression-free and overall survival rates were 80% (95% CI 65–89) and 94% (95% CI 82–98), respectively, and the reduced-dose radiotherapy was associated with a lower rate of acute, grade 3 dysphagia [31].

Despite the encouraging results from dose de-escalation studies, HPV-associated OPC does not have a uniformly favorable prognosis [33]. In RTOG 0129, Ang et al. demonstrated that the number of pack-years of tobacco smoking, tumor category, and nodal category all affected overall survival rates after radiotherapy, with more than 10 pack-years, larger tumors, and advanced adenopathy portending worse prognosis [34]. Although a pathologic complete response rate of 98% at the primary site after reduced-dose, definitive, chemoradiotherapy to 60 Gy (as noted by Chera et al.) is acceptable, the pathologic complete response rate of 84% for involved lymph nodes suggests that further studies are needed on the clinical implications of different responses at primary and nodal sites after de-escalated doses. Also, the 2-year progression-free survival rate of 80% for complete responders in the ECOG-ACRIN E1308 trial and the relatively short median follow-up times of dose de-escalation studies in general leave doubt regarding long-term locoregional outcomes. Collectively, these findings suggest that caution should be applied before dose de-escalation strategies are widely adopted in clinical practice.

## Proton therapy

**Fig. 1:**
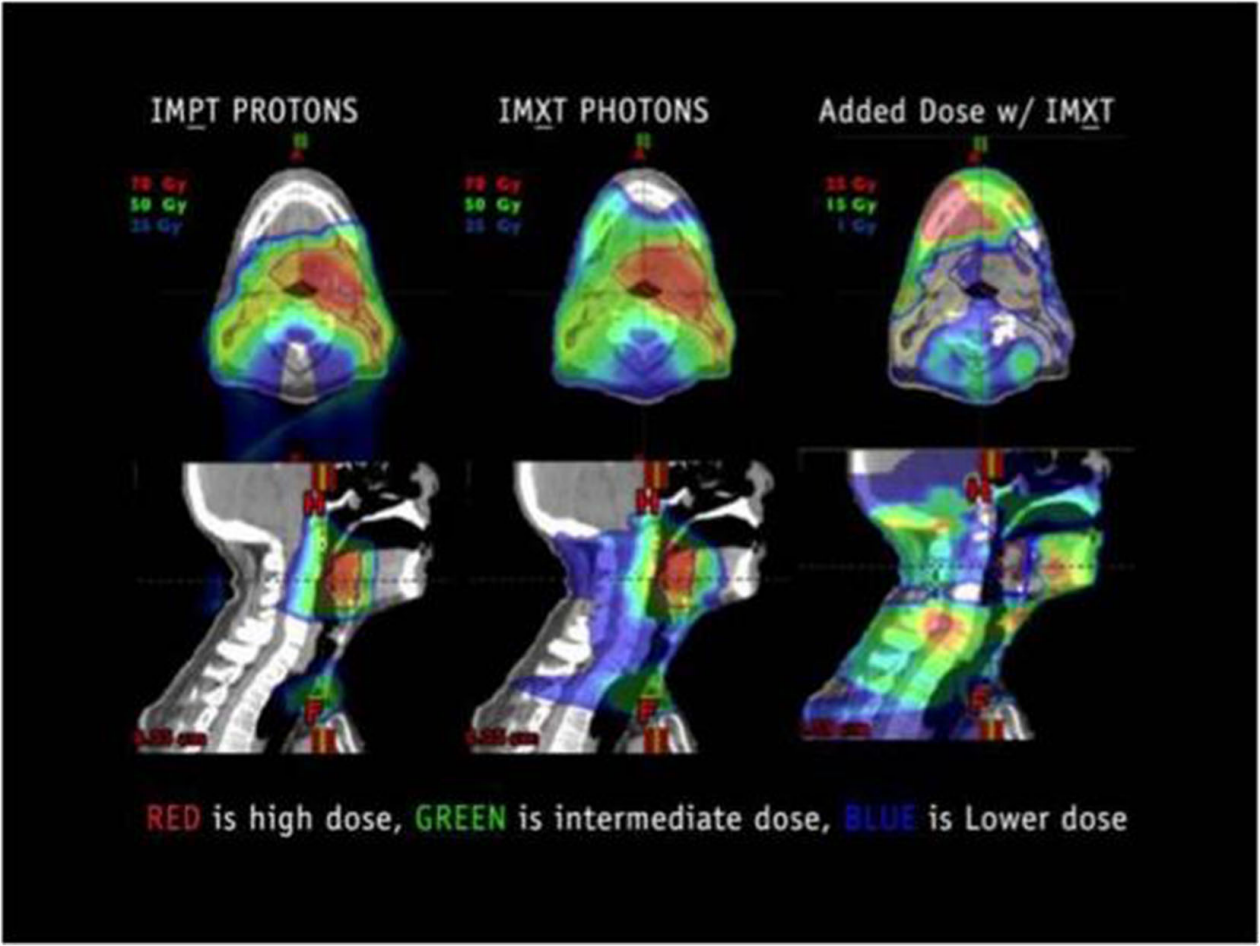
Coronal (top) and sagittal (bottom) views of treatment plans used to assess dose distributions associated with intensity-modulated proton therapy (IMPT) (left) and intensity-modulated photon (X-ray) radiotherapy (IMXT) (middle). The images on the right illustrate the additional radiation dose associated with IMXT relative to IMPT. Reprinted from “Intensity Modulated Proton Therapy for Head and Neck Tumors: Gilding the Lily or Holy Grail?” by Steven J. Frank, *International Journal of Radiation Oncology Biology Physics*, volume 95, page no. 38, Copyright 2016, with permission from Elsevier.

Intensity-modulated proton therapy is an advanced form of EBRT that may afford patients with HPV-associated OPC both high rates of disease control and reduced treatment-related morbidity through treatment de-intensification and elimination of unnecessary collateral radiation without dose de-escalation [35]. For all X-ray (photon)-based EBRT treatment techniques (including 2D, 3D-CRT, IMRT, and VMAT), radiation dose is delivered to normal tissues along the entire path of the beam, including entrance and exit doses. In contrast, proton therapy deposits radiation dose in accordance with the Bragg peak, resulting in a lower entrance dose, delivery of the maximum dose in the target volume of interest, and sharp dose fall-off thereafter [36]. Combining beams of various energies results in complete coverage of the target volume and almost no radiation dose delivered distally (Fig. 1). Treatments can be delivered by using either a passive scatter or an IMPT technique (also referred to as “pencil beam scanning”), with the latter achieving greater dose conformality [37].

## Biological enhancement of proton therapy for HPV-positive squamous cell carcinoma

Proton radiation dose is defined in terms of relative biological effectiveness (RBE) or the ratio of X-rays to protons required to produce a defined biological endpoint [38]. Relative biological effectiveness takes into consideration radiation dose, radiation fractionation, tissue type, and the linear energy transfer (LET) of protons in tissue. The RBE of protons is estimated to be 1.1, reflecting enhanced biological responses relative to X-rays of the same dose [38].

To investigate differences in cellular damage and mechanisms of cell death after proton versus photon radiation in the context of HPV-positive squamous cell carcinoma, Wang et al. delivered 4 Gy (RBE) of protons or 4 Gy of X-ray radiation to HPV-positive and HPV-negative squamous cell carcinoma cell lines [39, 40]. Relative to the HPV-negative cell lines, clonogenic survival in the HPV-positive cell lines was lower at 10 and 17 days. When mechanisms of cell death were considered, proton radiation caused more mitotic catastrophe and larger percentages of senescent cells than did X-ray radiation, with the greatest increase in the percentage of senescent cells occurring in an HPV-positive cell line at 6 days [40].

Wang and colleagues subsequently investigated differences in protein expression after proton or photon therapy and the use of niraparib, a poly ADP-ribose polymerase (PARP)-1/2 inhibitor that acts to block deoxyribonucleic acid (DNA) damage repair, as a radiosensitizer [41, 42]. At 24 h after irradiation, higher levels of proteins associated with DNA damage repair were expressed in cells irradiated with protons, and these changes were more profound for HPV-positive cells. At a cell survival fraction of 0.1, niraparib given with radiation increased the proton RBE by approximately 10% in HPV-positive cell lines compared with 3% in HPV-negative cell lines [42]. Although additional studies are needed, these findings suggest that HPV-associated OPC may be more sensitive to proton radiation, and they form the foundation for future research regarding the delivery of targeted, systemic therapies with proton therapy to enhance its RBE.

## Proton therapy treatment planning

Although a fixed RBE value of 1.1 for protons is used for radiation treatment planning, evidence exists to suggest that the RBE of protons varies along the path of the beam, being highest (and having highest LET) for the last few millimeters of the Bragg peak [43]. Given this source of variability in RBE, accurate estimation of the proton-beam range during treatment planning is crucial to ensure that the greatest dose is deposited within the target volumes. The range of a proton beam in a patient is a function of its initial energy and the ability of the patient’s tissues to attenuate the proton, the so-called stopping power. The stopping power is estimated indirectly from the Hounsfield units (i.e., computed tomography (CT) numbers) of the various tissues through which the beam passes. Image distortion due to CT number uncertainty, as well as artifacts created by metal clips, dental fillings, and prostheses, can result in compromised stopping power calculations, inaccurate estimation of beam range, and deposition of high-dose radiation in normal structures [44].

In addition to accurate estimates of beam range, the clinical use of proton therapy requires careful consideration of uncertainties introduced by intra-treatment weight loss, tumor shrinkage, and differences in patient positioning that can influence the size, shape, and location of target volumes and normal tissues during treatment [45]. In a retrospective analysis of 19 patients with OPC treated with IMRT, van Kranen et al. co-registered daily cone-beam CT scans obtained to verify patient positioning to treatment-planning CT scans and performed volumetric comparisons between structures of interest [46]. Although the cone-beam CT scans showed little change in the primary tumor volume between treatment fractions, a 4% per week reduction was noted in the volume of the parotid glands and a 10% decrease was noted in the lymph node clinical target volumes by week 5. Conversely, the pharyngeal constrictor muscles were found to increase in volume over the course of treatment. Because these changes have implications for radiation dosimetry—including coverage of target volumes, dose to normal tissues, radiation-related side effects, and locoregional disease control—the acquisition of verification CT scans with adaptive planning, as indicated, is necessary to ensure the fidelity of the treatment plans. Up to 40% of patients with HNC will require repeated planning as a result of anatomic changes noted on verification CT scans, and some patients will require several adaptive plans during the course of radiation treatment [47].

## Proton therapy for oropharyngeal cancer

The first hospital-based, clinical proton therapy facility in the USA opened in 1990 at Loma Linda University Medical Center in California. Nearly a decade passed before the inauguration of the second facility at Massachusetts General Hospital [48]. Despite the initially slow uptake of proton therapy, the number of proton therapy centers has proliferated quickly in the USA over the past decade, with 15 centers in operation in 2014 increasing to 34 centers in operation and two additional centers under construction in 2020 [49]. In the following sections, we highlight the investigational experience with proton therapy for OPC, from in silico studies to ongoing randomized trials comparing IMRT with IMPT for OPC.

### In silico studies

Cozzi et al. performed a dosimetric comparison of mixed photon-electron beam, 3D-CRT, IMRT, and proton plans for 5 HNC patients, including 3 cases of OPC, treated with 3D-CRT at the Oncology Institute of Southern Switzerland [50]. Except for the mixed photon-electron beam plan, all plans achieved comparable coverage of the target volume with 90% of the prescribed dose of 54 Gy (RBE). However, the proton plans were more favorable with respect to dose heterogeneity, maximum dose to the spinal cord, and dose received by two-thirds of the parotid glands (Table 1).

**Table 1:**
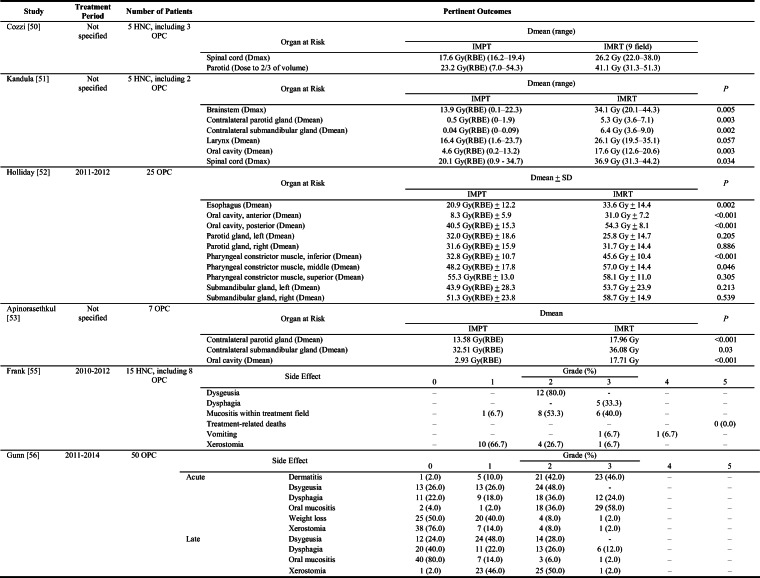
Dosimetric comparison of organs at risk for IMPT versus IMRT for HNC (Cozzi [50], Kandula [51], Holliday [52], Apinorasethkul [53]) and side effect rates after IMPT for HNC (Frank [55], Gunn [56])

Abbreviations: *HNC* head and neck cancer; *IMPT* intensity-modulated proton therapy; *IMRT* intensity-modulated photon (X-ray) radiotherapy; *OPC* oropharyngeal cancer; *RBE* relative biological effectiveness

Similarly, Kandula et al. reported a dosimetric comparison of IMRT and IMPT plans for 5 patients who had been treated with 60-66 Gy of IMRT for HNC at MD Anderson, including 2 patients with OPC [51]. Although both IMRT and IMPT plans showed adequate coverage of target volumes, the IMPT plans resulted in reduced radiation dose to the oral cavity, salivary structures, and brainstem. A subsequent MD Anderson dosimetric comparison between IMRT and IMPT plans for 25 patients with OPC who had been treated with 66-70 Gy (RBE) of IMPT reported lower mean doses to the anterior oral cavity (8.3 Gy (RBE) versus 31.0 Gy, *P*<0.001), posterior oral cavity (40.5 Gy (RBE) versus 54.3 Gy, *P*<0.001), inferior pharyngeal constrictor muscle (32.8 Gy (RBE) versus 45.6 Gy, *P*<0.001), and middle pharyngeal constrictor muscle (48.2 Gy (RBE) versus 57.0 Gy, *P*=0.046) with IMPT (Table 1) [52].

Apinorasethkul and colleagues compared treatment plans for IMRT versus IMPT for 7 patients with HPV-associated OPC who received IMPT postoperatively to doses of 60-63 Gy (RBE) [53]. All plans met planning parameters, including coverage of 95% of the target volume with the prescribed dose. However, proton plans were notable for lower mean doses to the oral cavity (2.93 Gy (RBE) versus 17.71 Gy, *P*<0.001), contralateral parotid gland (13.58 Gy (RBE) versus 17.96 Gy, *P*<0.001), and contralateral submandibular gland (32.51 Gy (RBE) versus 36.08 Gy, *P*=0.03).

### Case series

Loma Linda University Medical Center reported its early experience with proton therapy for 29 patients with stage III-IV OPC treated between October 1991 and June 2002 [54]. Patients were given 50.4 Gy of photon radiation followed by a concomitant boost of 25.5 Gy (RBE) delivered twice a day during the last 3.5 weeks of treatment, for a total tumor dose of 75.9 Gy. At a median follow-up time of 28 months (range 2–96 months), the 2-year rate of locoregional control was 93%.

In 2013, Frank and colleagues reported the first clinical experience with the use of multifield-optimized IMPT for treating 15 patients with HNC, including 8 patients with OPC, at MD Anderson [55]. Patients received 66-70 Gy (RBE) of radiation (with or without concurrent chemotherapy) as definitive therapy, after surgery, or after induction chemotherapy. Regarding acute side effects, xerostomia was reported by all patients, but was severe (grade 3) in only 1 patient. Similarly, all patients experienced mucositis in the treatment field, including 6 patients with grade 3 symptoms (Table 1). However, no patients reported grade 2 or worse anterior oral mucositis. Two patients required placement of a feeding tube for grade 3 dysphagia, and 80% of patients reported grade 2 dysgeusia. At a median follow-up interval of 28 months (range 20–35 months), the overall clinical complete response rate was 93.3% (95% CI 68.1–99.8).

Gunn and colleagues subsequently reported clinical outcomes for 50 patients with OPC treated with 66-70 Gy (RBE) of IMPT at MD Anderson from 2011 through 2014 [56]. Acute side effects included dermatitis, mucositis, xerostomia, and dysphagia. Acute grade 3 dysphagia occurred in a quarter of patients, and 20% of patients experienced inpatient hospitalization for poorly controlled pain, odynophagia, and dehydration secondary to oral mucositis. Twelve patients had feeding tubes placed (11 during treatment), and 5 of those patients required feeding tube nutrition for more than 3 months after completion of treatment. At a median follow-up time of 29 months (range 8–49 months), the 2-year overall survival rate was 94.5% (95% CI 81.4–98.5) and the 2-year progression-free survival rate was 88.6% (95% CI 75.8–95.1). An updated report of 103 patients with OPC treated from 2012 through 2016 showed that at a median follow-up time of 3.3 years (range 0.5–7.0 years), the 3-year overall survival, locoregional control, and disease-free survival rates were 96%, 93%, and 93%, respectively [57].

Aljabab et al. described the University of Washington Medical Center institutional experience with using IMPT to treat 46 patients with locally advanced OPC from March 2015 to August 2017 [58]. Patients received 70–74.4 Gy (RBE) definitively or 60–66 Gy (RBE) postoperatively. Acute grade 3 toxicities included dermatitis (76.1%), mucositis (71.7%), and xerostomia (6.5%). One patient experienced weight loss of more than 10% from baseline and 2 patients were hospitalized for pain and dehydration. Feeding tubes were placed in 18 patients (39.1%), with most (*n*=14) inserted before the initiation of radiotherapy. No patient experienced late grade 3 or higher xerostomia or dysgeusia, and only 1 patient experienced late grade 3 or higher dysphagia (Table 2). At a median follow-up time of 19.2 months (interquartile range 11.2-28.4 months), rates of progression-free survival and overall survival were 93.5% and 95.7%, respectively.

**Table 2:**
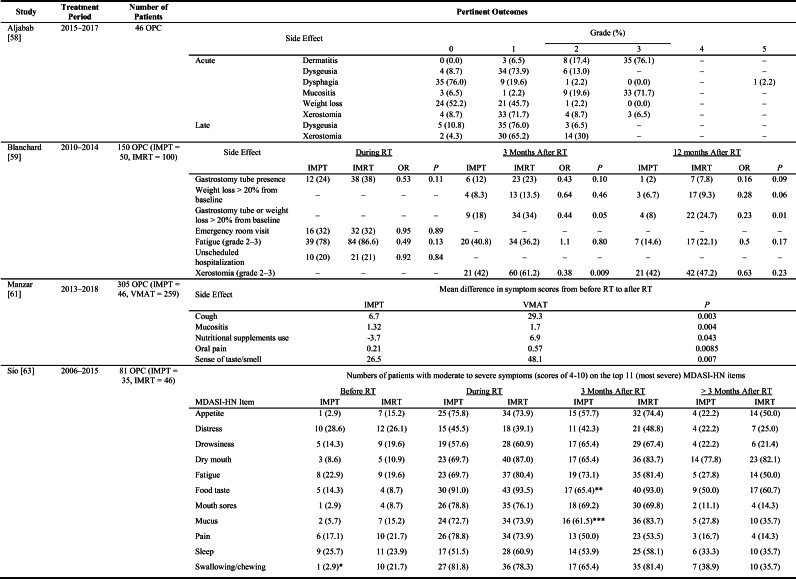
Side effect rates after IMPT for OPC (Aljabab [58]) and side effect comparison of IMPT versus IMRT/VMAT for OPC (Blanchard [59], Manzar [61], Sio [63])

Abbreviations: *IMPT* intensity-modulated proton therapy; *IMRT* intensity-modulated photon (X-ray) radiotherapy; *MDASI-HN* MD Anderson Symptom Inventory-Head and Neck; *OPC* oropharyngeal cancer; *RT* radiotherapy; *VMAT* volumetric arc radiotherapy

*P =0.014; **P =0.003; ***P=0.038

### Case-control studies

In a case-control series, Blanchard and colleagues compared clinical outcomes of 150 patients with OPC treated at MD Anderson from 2010 through 2014, 50 with IMPT and 100 with IMRT [59]. Patients received 70 Gy concurrent with chemotherapy or 66 Gy definitively. Cases were matched in 1:2 fashion based on laterality of treatment, OPC subsite, HPV status, disease stage, receipt of concurrent chemotherapy, and smoking status. Analysis of side effects revealed less severe xerostomia at 3 months after IMPT versus IMRT (odds ratio (OR) = 0.38, 95% CI 0.18–0.79, *P*=0.009). At 12 months’ follow-up time, rates of feeding tube placement or weight loss of more than 20% from pretreatment baseline also favored IMPT (OR = 0.23, 95% CI 0.07–0.73, *P*=0.01). At a median follow-up time of 29 months (range 8-49 months), the 3-year locoregional control rate and the 3-year distant control rate with IMPT were 91.0% and 97.8%, respectively. No significant difference was found in locoregional control or distant control between patients given IMPT versus those given IMRT.

In a study of osteoradionecrosis among 584 OPC patients treated with IMRT (*n* = 534) or IMPT (*n* = 50) at the same institution during an overlapping time interval, Zhang et al. found that, although no difference was observed between the two modalities with respect to the maximum dose to the mandible, the volume of the mandible receiving 5–70 Gy and the minimum, median, and mean doses were lower all with IMPT [60]. Forty-one patients treated with IMRT (7.7%) and 1 patient treated with IMPT (2.0%) developed osteoradionecrosis, with a median time to development of 11.4 months (range 6.7-16.1 months). All osteoradionecrosis developed in regions of the mandible that received at least 50 Gy, and the volume of the mandible that received 45–70 Gy was significantly associated with osteoradionecrosis (*P*<0.003).

Manzar et al. compared acute toxicities and patient-reported outcomes for 305 OPC patients treated with VMAT (*n* = 259) or IMPT (*n* = 46) at the Mayo Clinic from 2013 to 2018 with either 70 Gy definitively or 60–66 Gy postoperatively [61]. Analysis of feeding tube rates during and within 30 days of radiotherapy completion demonstrated lower use among patients treated with IMPT (19.6% versus 46.3%, OR = 0.27, 95% CI 0.12–0.59, *P*=0.001). Patients treated with IMPT also experienced lower rates of acute hospitalization (OR = 0.21, 95% CI 0.07–0.6, *P*=0.009). When the mean scores for measures of oral pain, mucositis, cough, sense of taste/smell, and use of nutritional supplements at end of treatment were compared with those at baseline, the differences in scores all favored IMPT (Table 2). At median follow-up times of 30 months for patients treated with VMAT and 12 months for those treated with IMPT, the 1-year rates of overall survival were 91.3% for VMAT and 92.6% for IMPT (*P*=0.98).

The symptom burden associated with treatment, rated according to the MD Anderson Symptom Inventory for HNC (MDASI-HN), was reported for patients with OPC who received concurrent chemoradiation at MD Anderson from 2006 through 2015 with either IMRT (*n* = 46) or IMPT (*n* = 35) [62, 63]. Differences in proportions of patients experiencing decreased appetite favored IMPT at two intervals after treatment, first within 3 months of treatment completion (MDASI-HN average score 4.68 versus 6.37, *P*=0.048) and later at more than 3 months after treatment completion (MDASI-HN average score 2.12 versus 4.14, *P*=0.036). Symptom burden associated with changes in taste also favored IMPT within 3 months of treatment (MDASI-HN average score 5.76 versus 7.70, *P*=0.01), but differences in this measure became nonsignificant during longer follow-up (Table 2). No significant difference was observed any of the other top 11 symptom scores (dry mouth, fatigue, pain, mucus, sleep, mouth sores, drowsiness, distress, swallowing) at baseline or follow-up. However, the average symptom burden for the top 5 symptom scores within 3 months of treatment completion favored IMPT (MDASI-HN average 5.15 versus 6.58, *P*=0.013).

### Randomized controlled trial

The “Randomized Trial of IMPT versus IMRT for the Treatment of Oropharyngeal Cancer of the Head and Neck” (NCT01893307) is the first prospective, phase II/III randomized trial to compare IMPT with IMRT for the treatment of OPC [64••]. The primary endpoint of the phase II study was the rate of grade 3 or higher treatment-associated side effects at 2 years. However, because these are physician-reported outcomes and side effects are often experienced differently by patients, this endpoint was deemed to lack objectivity and sensitivity. The primary endpoint was changed for the phase III trial to progression-free survival at 3 years, with secondary endpoints of physician-graded side effects and patient-reported outcome measures [55]. The non-inferiority, phase III design of this trial incorporates a measure of efficacy of IMPT and is consistent with the design of RTOG 1016 [10]. A cost-effectiveness analysis is also planned to clarify the overall value of proton therapy in light of its higher cost of delivery.

The “TOxicity Reduction using Proton bEam therapy for Oropharyngeal cancer (TORPEdO)” trial is the second, multicenter, phase III study of IMRT versus IMPT for OPC [65]. The primary endpoints of the trial are patient-reported outcomes, as measured by the University of Washington physical toxicity composite score, and feeding tube dependence or severe weight loss at 12 months after treatment completion. Like the “Randomized Trial of IMPT versus IMRT for the Treatment of Oropharyngeal Cancer of the Head and Neck,” the results of “TORPEdO” will include a cost-effective analysis. Enrollment at participating centers in the UK commenced in January 2020.

## Conclusions

In contrast to the overall incidence of HNC, the incidence of HPV-associated OPC has increased substantially over the past 2 decades. Its diagnosis in relatively young patients and its favorable prognosis have led to the development of treatment de-intensification strategies for HPV-associated OPC, including radiation dose de-escalation, in effort to reduce the likelihood of treatment-related side effects. However, because locoregional recurrence remains a source of treatment failure in HPV-associated OPC, the need persists for treatment techniques that permit treatment de-intensification to normal tissues without dose de-escalation to tumors. With appropriate patient selection, the physical and radiobiological properties of protons and the preclinical evidence demonstrating greater sensitivity of HPV-positive squamous cell carcinoma to proton radiation make proton therapy well suited to achieve this goal. The international experience with proton therapy suggests that, when compared with X-ray-based treatment, use of proton therapy for HPV-associated OPC confers comparable rates of disease control and reductions in both physician and patient-reported side effects. Given the inherent limitations of preclinical and retrospective studies of treatment for HPV-associated OPC, two trials currently underway, the “Randomized, Trial of IMPT versus IMRT for the Treatment of Oropharyngeal Cancer of the Head and Neck” and the “TOxicity Reduction using Proton bEam therapy for Oropharyngeal cancer (TORPEdO),” are expected to provide level 1 evidence regarding the indications for and value of proton therapy [65, 66].
